# A Glimpse into the Satellite DNA Library in Characidae Fish (Teleostei, Characiformes)

**DOI:** 10.3389/fgene.2017.00103

**Published:** 2017-08-14

**Authors:** Ricardo Utsunomia, Francisco J. Ruiz-Ruano, Duílio M. Z. A. Silva, Érica A. Serrano, Ivana F. Rosa, Patrícia E. S. Scudeler, Diogo T. Hashimoto, Claudio Oliveira, Juan Pedro M. Camacho, Fausto Foresti

**Affiliations:** ^1^Department of Morphology, Institute of Biosciences, São Paulo State University Botucatu, Brazil; ^2^Departamento de Genética, Facultad de Ciencias, Universidad de Granada Granada, Spain; ^3^CAUNESP, São Paulo State University Jaboticabal, Brazil

**Keywords:** concerted evolution, repetitive DNA, *in situ* hybridization, satellite DNA, genome evolution

## Abstract

Satellite DNA (satDNA) is an abundant fraction of repetitive DNA in eukaryotic genomes and plays an important role in genome organization and evolution. In general, satDNA sequences follow a concerted evolutionary pattern through the intragenomic homogenization of different repeat units. In addition, the satDNA library hypothesis predicts that related species share a series of satDNA variants descended from a common ancestor species, with differential amplification of different satDNA variants. The finding of a same satDNA family in species belonging to different genera within Characidae fish provided the opportunity to test both concerted evolution and library hypotheses. For this purpose, we analyzed here sequence variation and abundance of this satDNA family in ten species, by a combination of next generation sequencing (NGS), PCR and Sanger sequencing, and fluorescence *in situ* hybridization (FISH). We found extensive between-species variation for the number and size of pericentromeric FISH signals. At genomic level, the analysis of 1000s of DNA sequences obtained by Illumina sequencing and PCR amplification allowed defining 150 haplotypes which were linked in a common minimum spanning tree, where different patterns of concerted evolution were apparent. This also provided a glimpse into the satDNA library of this group of species. In consistency with the library hypothesis, different variants for this satDNA showed high differences in abundance between species, from highly abundant to simply relictual variants.

## Introduction

Eukaryotic genomes are composed of huge amounts of highly dynamic repetitive DNA sequences that may be dispersed throughout the genomes, e.g., transposable elements, or tandemly repeated, such as multigene families or satellite DNA (satDNA; Charlesworth et al., 1994; Jurka et al., 2005). satDNA constitutes a non-coding fraction of the genome, consisting in long arrays of tandemly repeated sequences, preferentially located on the heterochromatin of pericentromeric and subtelomeric chromosome regions, although their presence in euchromatic regions has already been reported (López-Flores and Garrido-Ramos, 2012; Plohl et al., 2012; Garrido-Ramos, 2015; Ruiz-Ruano et al., 2016). In general, satDNA sequences constitute different families that vary in localization, constitution, unit size and abundance (Garrido-Ramos, 2015). Since these sequences are highly dynamic genomic segments being susceptible to quick changes, these elements are generally species- or genus-specific (Vicari et al., 2010; Garrido-Ramos, 2015). According to the “library hypothesis” specific groups of related organisms share a common library of satDNAs that might be independently amplified in those distinct genomes (Fry and Salser, 1977). Such events might cause rapid changes in satDNA distribution and abundance profiles, even in closely related species (Plohl et al., 2012).

Up to now, most studies of satDNAs in fish genomes have focused on the development of chromosomal markers for evolutionary studies on B and sex chromosomes (Mestriner et al., 2000; Jesus et al., 2003; Vicari et al., 2010; Utsunomia et al., 2016). However, the evolutionary trends of satDNAs in closely related fish species have not yet been well evaluated, mainly if we consider that almost all discovered satDNA analyzed until now seemed to represent species- or genus-specific sequences (Garrido-Ramos et al., 1999; Leclerc et al., 1999; de la Herrán et al., 2001; Lanfredi et al., 2001; Robles et al., 2004; Martins et al., 2006).

Next generation sequencing (NGS) has been extensively used for several applications, including the in-depth characterization of satDNA sequences by similarity-based read clustering (Macas et al., 2011; Ruiz-Ruano et al., 2016). Such strategy has been frequently used for *de novo* characterization of repetitive DNA sequences in different organisms (Novák et al., 2010; Macas et al., 2011; Pagán et al., 2012; Camacho et al., 2015; García et al., 2015; Ruiz-Ruano et al., 2016; Utsunomia et al., 2016). In a recent study, Utsunomia et al. (2016) used graph-based clustering of sequence reads and isolated seven satDNAs (MS1-MS7) from the characid fish *Moenkhausia sanctaefilomenae*, two of which (MS3 and MS7) were fully characterized and mapped on chromosomes to unveil B chromosome origin in this species. More recently, it was evidenced that one of these satellites, MS1 satDNA (from now on referred to as MsaSat01-177, to follow the nomenclature rules suggested in Ruiz-Ruano et al., 2016), was found in the genomes of other characid fishes, such as *Astyanax paranae* and *A. mexicanus* (Silva et al., submitted), indicating its intergenera conservation and thus providing an interesting opportunity to investigate the evolutionary dynamics of this satellite in closely related species within Characidae.

Characidae is the largest family of freshwater fishes and comprises more than 1000 species (Eschmeyer and Fong, 2017). The phylogenetic relationships of this family are highly controversial and several species were considered *incertae sedis* by different authors (Javonillo et al., 2010; Oliveira et al., 2011; Thomaz et al., 2015). During the last few years, different studies using morphological and molecular evidence showed that Characidae is a well supported group which is subdivided into three different monophyletic clades (clades A, B, and C) (Weitzman and Malabarba, 1998; Javonillo et al., 2010; Oliveira et al., 2011; Thomaz et al., 2015). However, phylogenetic hypotheses within each of these clades are still scarce or unavailable and many genera are suspected to be non-monophyletic (Thomaz et al., 2015; Rossini et al., 2016).

Likewise, numerous cytogenetic studies were performed in representatives of this family during the last decades, which revealed extensive karyotype diversification at intra- and inter-species levels, including changes in diploid numbers, differential chromosomal location of multigene families and multiple origins of supernumerary chromosomes (Oliveira et al., 2009; Arai, 2011). However, the absence of satDNAs shared among species has impeded testing the main evolutionary hypotheses on this kind of repetitive DNA, such as concerted evolution and the library hypothesis (see above). Our main purpose here was to test these hypotheses on a satDNA shared between several Characidae species, using a combination of novel (Illumina sequencing) and traditional (PCR amplification, cloning, Sanger sequencing and FISH) approaches, in 10 species of Characidae fish belonging to A, B, and C clades. Therefore, our main objectives were: (i) delimiting the taxonomic spread of this satellite, (ii) comparing its chromosome abundance and localization between species, and (iii) investigating intra- and interespecific variation of MsaSat01-177 at nucleotide and chromosomal levels. All this information provided new insights on concerted evolution and the library hypothesis.

## Materials and Methods

### Ethics Statement

Sampling was carried out on private lands and the owners gave permission to conduct this study. The animals were captured using nets, transported to the Laboratory, kept in a fish tank and were anesthetized before the analyses. The animals were collected in accordance with Brazilian environmental protection legislation (Collection Permission MMA/IBAMA/SISBIO—number 3245) and the procedures for sampling, maintenance and analysis of the fishes were performed in compliance with the Brazilian College of Animal Experimentation (COBEA) and was approved (protocols 405 and 504) by the BIOSCIENCE INSTITUTE//UNESP ETHICS COMMITTEE ON THE USE OF ANIMALS (CEUA).

#### Sampling, Chromosomal Preparations and DNA Extraction

**FIGURE 1:**
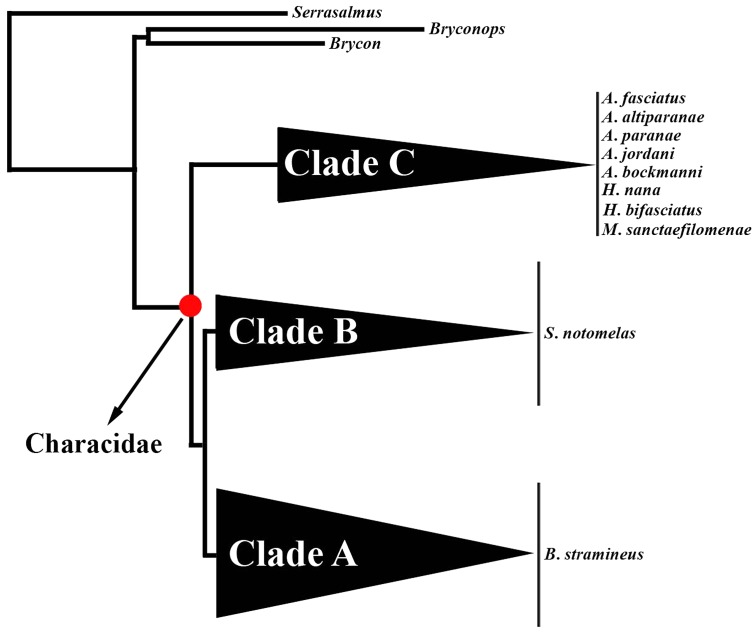
Phylogeny showing the relationships between clades “A,” “B,” and “C” of Characidae adapted from Thomaz et al. (2015). The red circle on the internal branches indicates Characidae. Species analyzed here from the three clades are indicated on the right.

In the present study, we analyzed ten allopatric Characidae species, namely *Astyanax paranae*, *A. bockmanni*, *A. altiparanae*, *A. fasciatus*, *A. jordani*, *M. sanctaefilomenae*, *Hasemania kalunga* and *Hyphessobrycon bifasciatus*, all of them belonging to clade C. In addition, *Bryconamericus stramineus* and *Serrapinus notomelas*, classified as clades A and B, respectively, were also analyzed (**Table 1**). The relationship between clades A, B, and C is represented in **Figure 1**. The available internal relationships among clade A species were not considered in this study, as several genera appear to be non-monophyletic. Cell suspensions from all species were already available in our laboratory from previous studies (Silva et al., 2013, 2014, 2016; Utsunomia et al., 2016), except for *H. bifasciatus*, *H. kalunga*, *B. stramineus* and *S. notomelas* whose karyotypes were analyzed here for the first time. Metaphase chromosomes were obtained from cell suspensions of the anterior kidney, according to Foresti et al. (1981). Genomic DNA was extracted from muscle or liver, using the Wizard Genomic DNA Purification Kit (Promega), following the manufacturer’s instructions.

**Table 1:** Analyzed species in the present study and information regarding MsaSat01-177 distribution patterns.

Clade	Species	2n	PCR	Pattern	Sites
Clade C	*M. sanctaefilomenae*	50	+	c	36
Clade C	*A. paranae*	50 + B	+	c	10
Clade C	*A. fasciatus*	46 + B	+	nc	nc
Clade C	*A. jordani*	50	+	c	18
Clade C	*A. altiparanae*	50	+	c	10
Clade C	*A. bockmanni*	50	+	c	2
Clade C	*Hasemania kalunga*	50	+	c	2
Clade C	*Hyphessobrycon bifasciatus*	50	+	c	18
Clade A	*B. stramineus*	52	-	-	-
Clade B	*S. notomelas*	52	-	-	-


*2n, Diploid chromosome number. Sites, number of chromosomes showing the satDNA. c, clustered; nc, non-clustered.*

### Whole-Genome Sequencing and Characterization of Monomers from Raw Reads

MsaSat01-177 was previously discovered in the *M. sanctaefilomenae* genome using RepeatExplorer (Utsunomia et al., 2016). Here, in order to perform a thorough search for MsaSat01-177 monomers in different genomic libraries, we used gDNA Illumina HiSeq2000 reads (2x101bp) from *M. sanctaefilomenae* and *A. paranae* stored in SRA (accession numbers SRR5839692 and SRR5461470, respectively). In addition, two individuals of *A. fasciatus* were sequenced on the Illumina MiSeq, yielding 2 × 250 bp paired-end reads. Firstly, in these three species, we performed a random sequence subsampling step of 5.000.000 paired-end reads per species. Detailed information about the used Illumina libraries is shown in **Table 2**. In addition, we used other gDNA Illumina MiSeq reads (2 × 250 bp) recently sequenced in our laboratory (data not shown) from several characiform fishes, including Anostomidae (*Megaleporinus macrocephalus* and *Leporinus friderici*), Crenuchidae (*Characidium gomesi*) and Serrasalmidae (*Piaractus mesopotamicus*), all belonging to the Characiformes order (Oliveira et al., 2011).

**Table 2:** Genetic variation found in the monomers of MsaSat01-177 extracted from Illumina reads of three different species and PCR-amplified from other five different characid species.

Species	Library	Seq	*N*	Size	Hap	Hd	π
*M. sanctaefilomenae*	2 × 101 bp	10^6^	470	172–180bp	84	0.884	0.013
*A. paranae*	2 × 101 bp	10^6^	201	170–180bp	68	0.9844	0.055
*A. fasciatus*	2 × 250 bp	10^6^	8	175–178bp	8	1	0.18


*Seq, Number of sequences in the primary fastq library. N, number of isolated monomers. Hap, number of haplotypes. Hd, haplotype diversity. π, nucleotide diversity.*

To obtain a detailed and reliable score of haplotype abundance for MsaSat01-177 sequences from the genomic libraries of *A. paranae*, *A. fasciatus* and *M. sanctaefilomenae*, we extracted complete monomers directly from the Illumina raw reads, as this is expected to provide accurate estimates of haplotype abundance without the bias of PCR amplification. For this purpose, we performed a series of bioinformatic workflows that included joining the paired-end reads, aligning them against the MsaSat01-177 sequence and trimming the ends to get full monomers, as described in Utsunomia et al. (2016). Importantly, singletons (e.g., sequence variants found only once) were discarded at this stage of the analysis in order to minimize the impact of possible sequencing errors. Collected monomers from Illumina reads in the three species were aligned separately using the Muscle algorithm (Edgar, 2004), under default parameters, to be displayed as sequence logos using the WebLogo 3.3 software (Crooks et al., 2004). The obtained monomers were used for all downstream analyses in this study, except for RepeatExplorer (described below).

In order to investigate possible structural variation of MsaSat01-177 in these three species and to search for possible associations with other repetitive elements, we selected pairs of reads showing homology with this satDNA in each gDNA library separately, by using BLAT (Kent, 2002). This step is implemented in a custom script^1^. We then used the selected read pairs from each library to run RepeatExplorer clustering (Novák et al., 2013) with at least 2 × 2500 reads.

### satDNA Amplification, Cloning and Sequencing

After complete characterization of the repetition unit of MsaSat01-177, different sets of divergent primer pairs were designed: MsaSat01F1 (5′-TTTTGACCATTCATGAAACCTTG-3′) and MsaSat01R1 (5′-ACCAGAATCACATACCGCGG-3′); MsaSat01F2 (5′-TGCCCATGCATTTTCCCACT-3′) and MsaSat01R2 (5′-GAARGATTTCATGAAATTTYGC-3′). PCR reactions were performed in 1x PCR buffer, 1.5 mM MgCl_2_, 200 μM each dNTP, 0.1 μM each primer, 2 pg–10 ng of DNA and 0.5 U of *Taq* polymerase (Invitrogen). The cycling program for amplification consisted of an initial denaturation at 95°C for 5 min, followed by 30 cycles at 95°C for 20 s, 63°C for 30 s, 72°C for 20 s and a final extension at 72°C for 15 min. The PCR products were visualized in 2% agarose gels, and the fragment obtained from each sample was extracted from the gel and cloned into the pGEM-T Easy Vector (Promega, Madison, WI, United States). DNA sequencing was performed with the Big Dye TM Terminator v3.1 Cycle Sequencing Ready Reaction Kit (Applied Biosystems) following the manufacturer’s instructions. Consensus sequences from forward and reverse strands of the sequenced clones were obtained using Geneious Pro v.8.04.

### DNA Probes and FISH

DNA probes for MsaSat01-177 were obtained by PCR amplification on genomic DNA from all species, except *B. stramineus* and *S. notomelas*, using the same conditions described above and labeling DNA with digoxigenin-11-dUTP or biotin-16-dUTP. Complementarily, probes were also obtained directly from single cloned sequences to compare the results. Thus, for every species, FISH was performed using probes obtained from their own genomes.

Fluorescence *in situ* hybridization was performed under high-stringency conditions using the method described by Pinkel et al. (1986). Pre-hybridization conditions included a 1-h incubation with RNAse (50 μg/ml) followed by chromosomal DNA denaturation in 70% formamide/2x SSC for 5 min at 70°C. For each slide, 300 μl of hybridization solution (containing 200 ng of labeled probe, 50% formamide, 2x SSC and 10% dextran sulfate) was denatured for 10 min at 95°C, then dropped onto the slides and allowed to hybridize overnight at 37°C in a moist chamber containing 2x SSC. Post-hybridization, all slides were washed in 0.2x SSC/15% formamide for 20 min at 42°C, followed by a second wash in 0.1x SSC for 15 min at 60°C and a final wash at room temperature in 4x SSC, 0.5% Tween for 10 min. Probe detection was carried out with avidin-FITC (Sigma) or anti-digoxigenin-rhodamine (Roche), and the chromosomes were counterstained with DAPI (4′,6-diamidino-2-phenylindole, Vector Laboratories) and analyzed under an optical photomicroscope (Olympus BX61). Images were captured with an Olympus DP70 digital camera and with the Image Pro plus 6.0software (Media Cybernetics). From each individual, a minimum of five cells was analyzed for FISH.

### Nucleotide Analyses

A global alignment from both Illumina-derived and PCR-derived sequences was generated using the Muscle algorithm (Edgar, 2004) under default parameters. DNA diversity analyses, considering indels and all haplotypes, were performed with DnaSP v5.05 (Librado and Rozas, 2009). In order to get fewer haplotypes in the Minimum spanning tree (MST), we performed a clustering analysis with CD-HIT-EST (Li and Godzik, 2006) selecting a sequence identity level of 99%. The MST was built on the basis of pairwise differences using ARLEQUIN v3.5.1.3 (Excoffier and Lischer, 2010) and was visualized with HAPSTAR (Teacher and Griffiths, 2011).

## Results

### Chromosomal Analysis

Cytogenetic analyses evidenced different diploid chromosome numbers for the analyzed species (**Table 1**). PCR amplification of MsaSat01-177 yielded a ladder pattern in 2% agarose gels for all species within clade C, while no visible banding patterns were detected for species from clade A (*B. stramineus*) and B (*S. notomelas*), suggesting that these sequences are not present in these species or were not amplified with the designed primers due to high sequence divergence. Also, FISH with inter-specific probes did not returned any visible signal on the chromosomes of these two species (data not shown).

**FIGURE 2:**
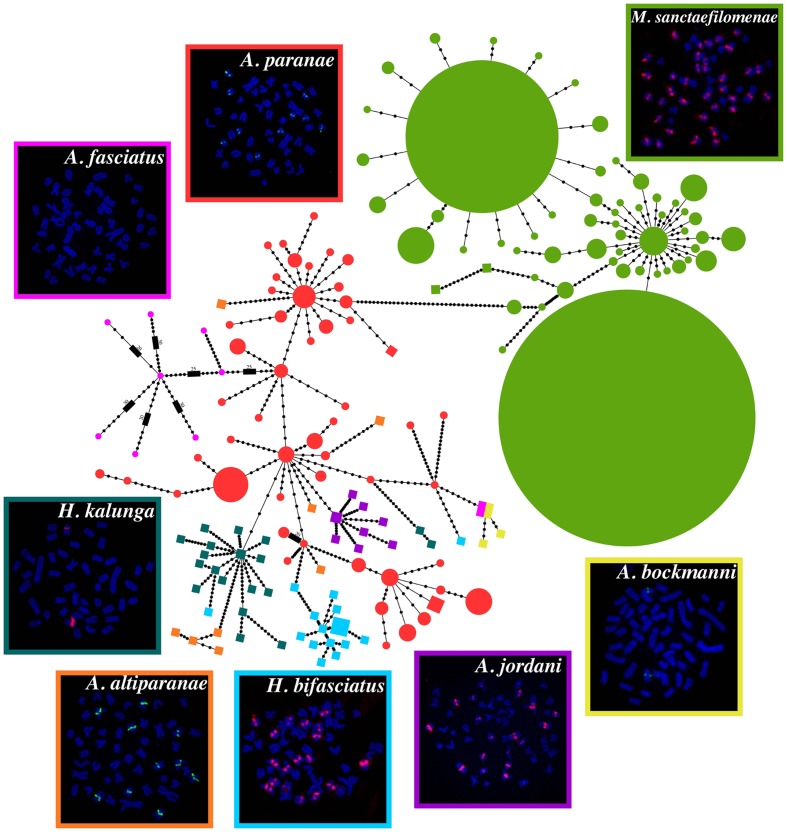
Minimum spanning tree (MST) showing the relationships between the different haplotypes of MsaSat01-177 obtained from distinct species. Colored circles represent haplotypes retrieved from Illumina reads, and the diameter of the circles is proportional to their abundance, whereas PCR-amplified haplotypes are represented by colored squares. Each black dot represents a mutational step. Metaphase plates after FISH with MsaSat01-177 probe are also shown and the colors of the borders correspond to the colors of the circles/squares.

FISH evidenced that MsaSat01-177 shows a non-clustered organization in *A. fasciatus*, but a clustered distribution in the other C-clade species. Remarkably, all clusters for this satDNA were located pericentromerically (**Figure 2**), but showing extensive variation among species concerning the number of chromosomes carrying it, namely two in *H. kalunga* and *A. bockmanni*, 10 in *A. altiparanae* and *A. paranae*, 18 in *H. bifasciatus* and *A. jordani*, and 36 in *M. sanctaefilomenae*.

### Bioinformatic and Molecular Analyses

Selection of Illumina reads showing homology with MsaSat01-177 resulted in 298.622, 3.160 and 24 reads in *M. sanctaefilomenae*, *A. paranae* and *A. fasciatus*, respectively. Those found in the latter species were insufficient for RepeatExplorer analysis and, in *A. paranae*, we had to use two copies of each read in order to meet the requirement of 5.000 reads minimum. Finally, for *M. sanctaefilomenae*, we subsampled the reads from 298.622 to 30.000 reads to optimize RepeatExplorer calculations. Output data evidenced spherical graphs for MsaSat01-177 in both species (**Supplementary Figure S1**), as expected for satDNAs. Although these results do not exclude the possibility of association with other repetitive sequences, they indicate that this satDNA is not primarily associated with other repetitive elements.

**FIGURE 3:**
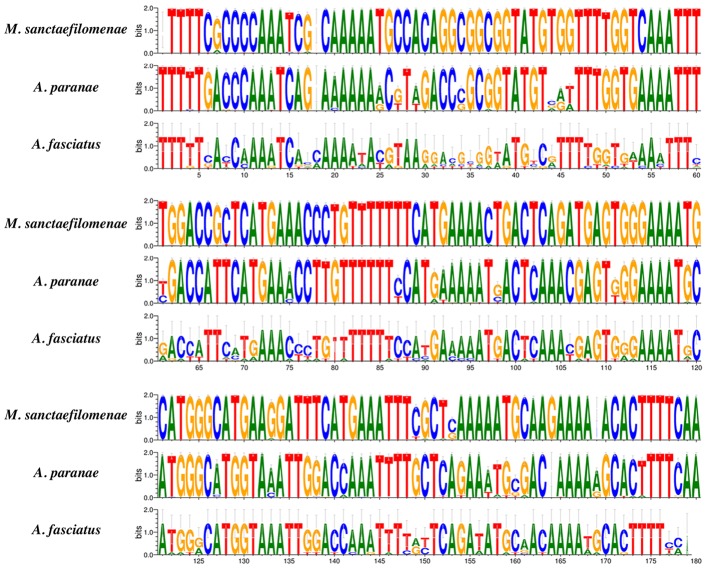
MsaSat01-177 sequence logos, where differences between consensus sequences obtained from *M. sanctaefilomenae*, *A. paranae* and *A. fasciatus* might be observed.

We successfully extracted monomers directly from sequencing reads of *A. paranae*, *A. fasciatus* and *M. sanctaefilomenae* and the detailed information is summarized in **Table 2**. Conversely, searches for MsaSat01-177 in distinct Characiformes genomes, except Characidae, did not yield any result, suggesting that MsaSat01-177 is not present in other families than Characidae within this order. In this context, we restricted our high-throughput analyses to the three Characidae fishes available. The extraction of MsaSat01-177 monomers from read pairs showing overlapping, resulted in a total of 470, 201 and 8 monomers in *M. sanctaefilomenae*, *A. paranae* and *A. fasciatus*, respectively. The eight sequences in the latter species showed the highest nucleotide diversity (π), whereas those in *A. paranae* showed higher nucleotide diversity than those in *M. sanctaefilomenae* (**Table 2**). Sequence logos corroborated this result and exhibited different levels of sequence conservation between the analyzed species for MsaSat01-177 monomers, with those in *M. sanctaefilomenae* showing higher conservation than those in *A. paranae* and *A. fasciatus* (**Figure 3**).

PCR amplification in the C-clade species, and subsequent cloning and sequencing, yielded several sequences per species (**Table 3**). Notably, most of the few sequenced clones in *A. paranae*, *A. fasciatus* and *M. sanctaefilomenae* were also found among the Illumina reads. In general, the number of haplotypes was almost equal to the number of sequenced clones for all species, while nucleotide diversity (π) values were variable, with those in *A. altiparanae* showing the highest values.

**Table 3:** Genetic variation found in the monomers of PCR-amplified MsaSat01-177 from eight different characid species.

Species	*N*	Size	Hap	Hd	π
*M. sanctaefilomenae*	2	179	2	1	0.06
*A. paranae*	3	177–178	2	0.66	0.07
*A. fasciatus*	2	167	1	0	0
*A. altiparanae*	8	177–186bp	8	1	0.086
*A. bockmanni*	5	167bp	4	0.9	0.01
*A. jordani*	9	177–182bp	9	1	0.03
*H. bifasciatus*	19	166–180bp	17	0.98	0.02
*H. kalunga*	19	177–178bp	18	0.99	0.05


*N, number of sequences. Hap, number of haplotypes. Hd, haplotype diversity. π, nucleotide diversity.*

**FIGURE 4:**
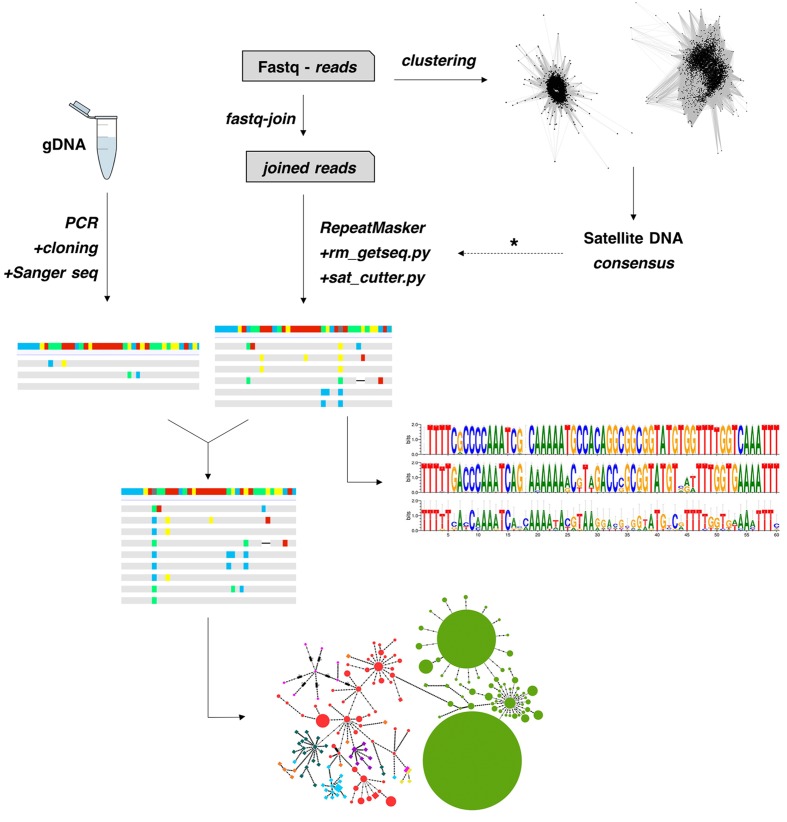
Pipeline used for satDNA analysis in this study. A typical clustering of raw reads with RepeatExplorer is performed to search for a consensus satDNA sequence. After that, this consensus sequence is used as reference (dotted arrow) to collect monomers directly from the reads using a series of custom python scripts. The isolated monomers are then analyzed for different features, such as intragenomic diversity and nucleotide analyses. Also, a strategy combining PCR, cloning and Sanger sequencing might be performed to isolate monomers if NGS reads are not available. Finally, a global alignment is then performed to construct minimum-spanning-trees.

In order to obtain a global alignment and generate a MST, we firstly performed a clustering step with CD-HIT-EST to the Illumina-derived monomers to reduce the numbers of haplotypes. Thus, a total of 470, 201 and 8 monomers were reduced to a final matrix with 55, 51 and 8 clusters, from *M. sanctaefilomenae*, *A. paranae* and *A. fasciatus*. After that, a final alignment matrix was composed of 150 haplotypes, 114 of which were obtained from Illumina reads and 36 from PCR clones. Considering this whole alignment, we built a MST, considering haplotype relative abundance, which evidenced overall species-specific groups of haplotypes, the main exception being *A. paranae* which showed several groups linked with those in most remaining species (**Figure 2**). The main steps performed in this study to obtain the described results are represented in **Figure 4**.

## Discussion

It is generally assumed that satDNA sequences evolve following a pattern of “concerted evolution,” as a consequence of intraspecific sequence homogenization and fixation (Dover, 1982, 1986). Notably, the homogenization process is driven by molecular mechanisms such as unequal crossing-over or gene conversion (Smith, 1976; Dover, 1986), which usually lead to a quite low sequence divergence among monomers within satDNA arrays (Plohl et al., 2012). Our sequence analysis of MsaSat01-177 in eight Characidae fish species has revealed some interesting features. First, we could indeed observe a higher homogenization within species since most haplotypes in **Figure 2**, coming from a same species, tended to group together, with the exception of those in *A. paranae*, which were distributed into three different groups. This is the expected pattern for concerted evolution of satDNA, but the *A. paranae* case demands additional explanations (see below).

Second, a comparison between the number of clusters observed by FISH and nucleotide diversity, in the three species analyzed by Illumina sequencing, revealed an interesting pattern, since the species showing the highest number of clusters (*M. sanctafilomenae*) showed the lowest nucleotide diversity, whereas the species that failed to show clusters (*A. fasciatus*) showed the highest diversity, with *A. paranae* showing intermediate values for both parameters. Population demographical events (e.g., bottlenecks) might have contributed to yield a pattern like this (Ardern et al., 1997; Pons et al., 2002), but between species differences in the homogenization/mutation balance could also provide an explanation (reviewed in Ugarković and Plohl, 2002). For example, a recent amplification event of the MsaSat01-177 in *M. sanctaefilomenae* could explain its low nucleotide diversity. On the other hand, differential amplification between satellite subfamilies could explain the high diversity in *A. paranae* (Willard and Waye, 1987).

Our present results have shown the presence of the MsaSat01-177 satDNA, previously described in the characid fish *M. sanctaefilomenae* (Utsunomia et al., 2016), in eight species belonging to four different Characidae genera, belonging to Clade C (Oliveira et al., 2011). Notably, we could neither amplify it by PCR in species belonging to clades A or B, nor find any trace of this sequence in genomic libraries of other Characiform families, suggesting that the MsaSat01-177 satDNA might be restricted to the C-clade species. This suggests the conservation of this satDNA in this fish group and allows testing some features of the satDNA library hypothesis (Fry and Salser, 1977) in these Characidae fish.

The MST shown in **Figure 2** provides a glimpse into a small part of the satDNA library of the Characid C-clade species, as it only shows some library volumes for the MsaSat01-177 satDNA in only eight species. Clearly, the complete library should include all haplotypes found in all species for the whole satellitome catalog, with MSTs for each satDNA family and some families connected by common branches if belong to a same superfamily. Of course, this appears to be an impossible task, but looking at a small corner of the library is also very illustrative. Firstly, **Figure 2** shows that species from four different genera share the MsaSat01-177 satellite, with *Astyanax paranae* showing connections with all remaining species, but with higher number of differences with *M. sanctafilomenae*. The central position of *A. paranae* among the five *Astyanax* species might actually be an artifact due to the higher number of sequences obtained from sequence reads in this species. However, in the case of *A. fasciatus*, we also employed this approach but we found only 24 reads showing homology with the MsaSat01-177 satellite, indicating that its presence in this species is just a relic, with only a few small arrays for eight highly divergent haplotypes scattered through the genome, since it was not apparent by FISH (see **Figure 2**). Ruiz-Ruano et al. (2016) suggested that satDNA follows a three-step evolutionary pathway: birth, dissemination and clustering. It is thus conceivable that, in *A. fasciatus*, this satDNA has not reached the third stage, and the extremely high divergence shown by the few units found (π = 0.18) suggests that they are not subjected to concerted evolution thus probably being disseminated across the genome. In high contrast, in *M. sanctafilomenae*, we found two extremely abundant haplotypes along with many other less abundant ones at few mutational steps, suggesting that sequence homogenization works very efficiently in this species, as also indicated by its low nucleotide diversity (π = 0.013). This indicates that this species has lost much of the satDNA variants, which were originally present in the common ancestor of the eight species here analyzed. The case of *A. paranae* is intermediate (π = 0.055), suggesting that it has preserved a higher proportion of the original satDNA variation, presumably because satDNA homogenization works poorer than in *M. sanctafilomenae*. This might explain the central position of *A. paranae* in the tree. As a whole, the former observations are consistent with the independent amplification of satDNA variants in different genomes, suggested by the library hypothesis (Fry and Salser, 1977).

The MST in **Figure 2** also suggests that the Illumina approach is much more informative than the PCR one and that the traditional conclusions on concerted evolution inferred from the latter method could be biased by the unavoidable filtering inherent to the PCR reaction, with products enriched in those sequences to which the primers are able to anchor. Illumina sequencing, however, provides a random sample of sequences thus giving more realistic information. The PCR bias might explain why in **Figure 2** the immense majority of haplotypes obtained by PCR were grouped per species. However, on the basis of the multiple connections shown by the Illumina haplotypes in *A. paranae*, we can imagine a much more intricate haplotype tree with connections between most species. Therefore, the analysis of satDNA variation through Illumina sequencing can open, in the next future, the satDNA library doors wide.

At genus level, it appears that the *Astyanax* satDNA library keeps more variation in common with those in *Hasemania* and *Hyphessobrycon* than with that in *Moenkhausia*, suggesting closer relationship between the three former genera. However, this might be a false impression due to the efficient homogenization in *M. sanctafilomenae*, which has erased, in the satDNA library, many signs of their common descent.

In general, satDNA accumulation in heterochromatic areas is an overall trend, although recent analyses have revealed that euchromatic areas might also be occupied by this kind of repetitive sequences (Kuhn et al., 2012; Ruiz-Ruano et al., 2016). Here, we found that all clusters of MsaSat01-177 found on the chromosomes of seven species were exclusively located on heterochromatic pericentromeric regions. The high differences between species for the number and size of MsaSat01-177 clusters, in the seven species where this satellite was visualized by FISH, from two in *A. bockmanni* and *H. kalunga* to 36 in *M. sanctafilomenae* (see **Table 1**), indicates that satDNA clustering has followed different evolutionary pathways in most species, although it is also conceivable that some clusters residing in chromosomes showing synteny among species might have descended from a common ancestor. According to the three-step hypothesis (Ruiz-Ruano et al., 2016), the former results suggest that satDNA evolution may follow different pathways in different species by reaching variable degrees of interchromosomal spread. Notably, *A. paranae* is phylogenetically more related to *A. bockmanni* than to *A. altiparanae* (Rossini et al., 2016) consistent with our MST. However, the number of sites per genome evidenced by FISH (10, 2 and 10, respectively) would not indicate that. In this context, as other repetitive DNA sequences, the number of sites and satDNA-bearing chromosomes do not appear to completely reflect phylogenetic relationships, and thus probably reflect historical contingency. Unfortunately, as mentioned before, a complete phylogeny considering the taxa sampled in our study is not available (Oliveira et al., 2011; Thomaz et al., 2015; Rossini et al., 2016).

As components of the repetitive fraction of genomes, satDNA is highly dynamic and its abundance might rapidly change due to expansion and/or decrease of these sequence arrays (Plohl et al., 2008; Garrido-Ramos, 2015). Therefore, different mechanisms may have led MsaSat01-177 to be highly abundant and homogenized in *M. sanctaefilomenae*, presumably due to recent amplification on 72% of its chromosomes (50 chromosomes – 36 FISH signals) (**Supplementary Figure S2**). In contrast, this satellite is relictual in *A. fasciatus*, it shows a cluster on a single chromosome pair in *A. bockmanni* and *H. kalunga*, or several chromosome pairs in the remaining species. Such dynamics, at the chromosomal level, has frequently been reported for several satDNA sequences in a wide range of organisms, at the intra- and interespecific levels (Plohl et al., 2012; Garrido-Ramos, 2015). Although multiple mechanisms have been put forward to explain this variation, such as unequal crossing-over, ectopic recombination, replication slippage, association with transposable elements and extrachromosomal circular DNA (Dover, 1993; McMurray, 1995; Hancock, 1996; Cohen et al., 2010; Milani and Cabral-de-Mello, 2014; Ruiz-Ruano et al., 2015, 2016), the ultimate explanation has not yet been figured out.

Taken together, our present results have provided evidence for the presence of a shared satDNA among several species within Characidae, which probably arose after the split of Clade C. The chromosomal distribution of MsaSat01-177 was highly variable and several spreading mechanisms might be acting in this case. As expected, monomers from all species are subjected to concerted evolution, except those in *A. fasciatus* where short tandem arrays of MsaSat01-177 are probably scattered across the genome. In addition, sequence homogenization levels were also different among species, and our results have also shown the differential amplification of some variants for this satellite in different species. This is in high consistency with the library hypothesis (Fry and Salser, 1977), that a same satellite family can follow different evolutionary pathways in different species, including not only for amplification levels but also for chromosome distribution.

## Author Contributions

RU, DS, PS, ES, and IR collected the samples, performed the cytogenetic analyses, the production of DNA probes and the cloning and FISH experiments. RU, DS, and FR-R performed the bioinformatics analyses. RU, DS, PS, ES, IR, and FR-R drafted the text and designed the figures. DH, JC, CO, and FF critically revised the manuscript and approved the final version.

## Conflict of Interest Statement

The authors declare that the research was conducted in the absence of any commercial or financial relationships that could be construed as a potential conflict of interest.

## Footnotes

1 https://github.com/fjruizruano/ngs-protocols/blob/master/mapping_blat_gs.py

## Supplementary Material

**FIGURE S1 |** Cluster-graphs obtained for MsaSat01-177 from *A. paranae* and *M. sanctaefilomenae* after analysis with RepeatExplorer.

**FIGURE S2 |** Metaphases from different Characidae species after FISH with MsaSat01-177 probes.
