# Supplementary figures and images for: A Glimpse into the Satellite DNA Library in Characidae Fish (Teleostei, Characiformes)
Source: Front Genet. 2017 Aug 14;8:103. doi: 10.3389/fgene.2017.00103 (PMC5557728; doi:10.3389/fgene.2017.00103)

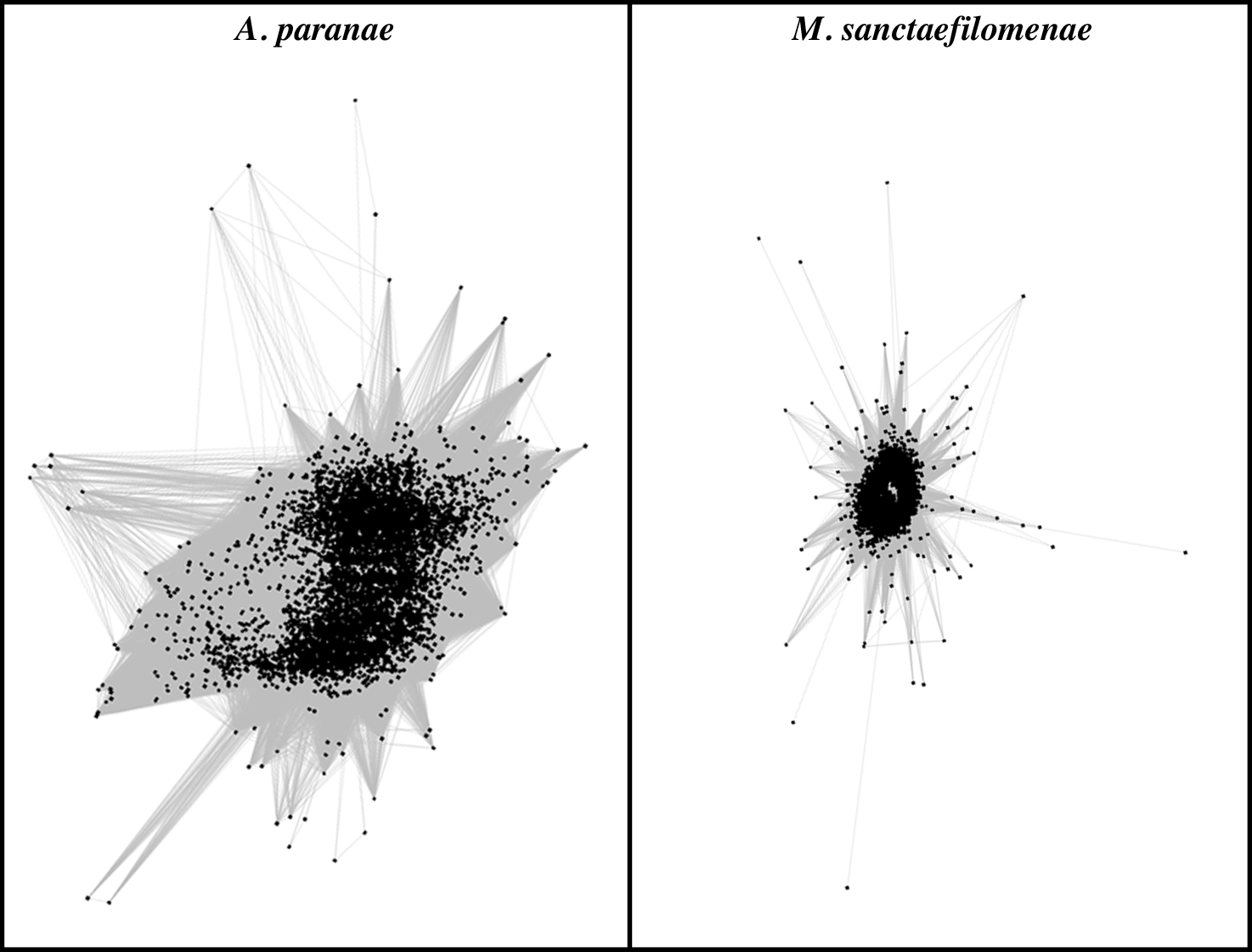

Supplement: Supplementary file 1 [file Image_1.TIF]

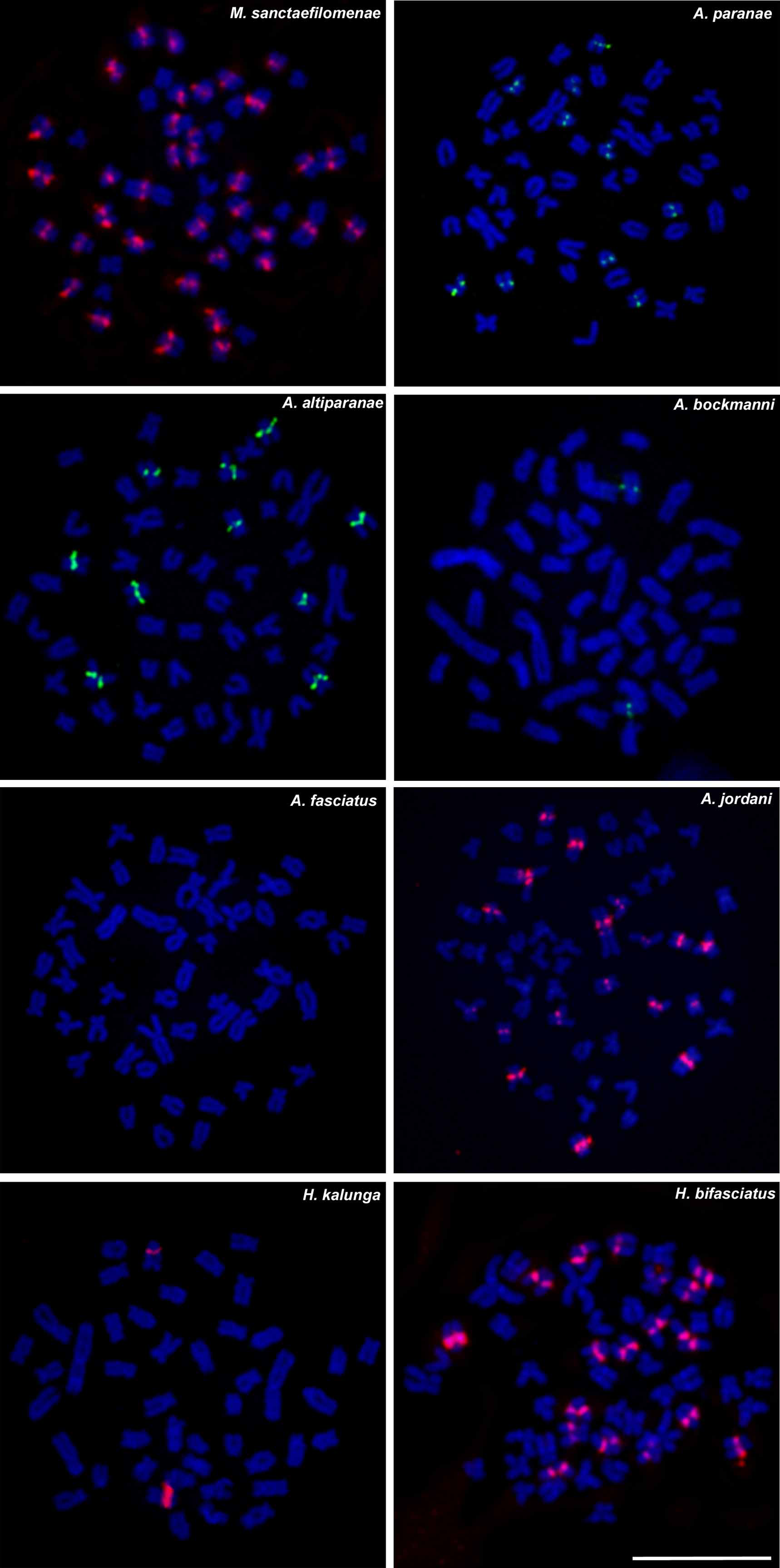

Supplement: Supplementary file 2 [file Image_2.TIF]
